# The Defense Response of *Nicotiana benthamiana* to Peanut Stunt Virus Infection in the Presence of Symptom Exacerbating Satellite RNA

**DOI:** 10.3390/v10090449

**Published:** 2018-08-23

**Authors:** Aleksandra Obrępalska-Stęplowska, Agnieszka Zmienko, Barbara Wrzesińska, Michal Goralski, Marek Figlerowicz, Joanna Zyprych-Walczak, Idzi Siatkowski, Henryk Pospieszny

**Affiliations:** 1 Department of Entomology, Animal Pests and Biotechnology, Institute of Plant Protection—National Research Institute, 20 Władysława Węgorka Street, 60-318 Poznań, Poland; b.wrzesinska@iorpib.poznan.pl; 2 Institute of Bioorganic Chemistry, Polish Academy of Sciences, 12/14 Noskowskiego Street, 61-704 Poznań, Poland; akisiel@ibch.poznan.pl (A.Z.); gurral@poczta.onet.pl (M.G.); marek.figlerowicz@ibch.poznan.pl (M.F.); 3 Institute of Computing Science, Faculty of Computing Science, Poznań University of Technology, 2 Piotrowo Street, 60-965 Poznań, Poland; 4 Department of Mathematical and Statistical Methods, University of Life Sciences in Poznań, Wojska Polskiego 28 Street, 60-637 Poznań, Poland; zjoanna@up.poznan.pl (J.Z.-W.); idzi@up.poznan.pl (I.S.); 5 Department of Virology, Institute of Plant Protection—National Research Institute, 20 Władysława Węgorka Street, 60-318 Poznań, Poland; h.pospieszny@iorpib.poznan.pl

**Keywords:** plant–virus interaction, satellite RNA, transcriptomics, leaf transcriptome, plant defense, stress response, phosphorylation, posttranscriptional gene silencing (PTGS)

## Abstract

Peanut stunt virus (PSV) is a widespread pathogen infecting legumes. The PSV strains are classified into four subgroups and some are defined by the association of satellite RNAs (satRNAs). In the case of PSV, the presence of satRNAs alters the symptoms of disease in infected plants. In this study, we elucidated the plant response to PSV-G strain, which occurs in natural conditions without satRNA. However, it was found that it might easily acquire satRNA, which exacerbated pathogenesis in *Nicotiana benthamiana.* To explain the mechanisms underlying PSV infection and symptoms exacerbation caused by satRNA, we carried out transcriptome profiling of *N. benthamiana* challenged by PSV-G and satRNA using species-specific microarrays. Co-infection of plants with PSV-G + satRNA increased the number of identified differentially expressed genes (DEGs) compared with the number identified in PSV-G-infected plants. In both treatments, the majority of up-regulated DEGs were engaged in translation, ribosome biogenesis, RNA metabolism, and response to stimuli, while the down-regulated DEGs were required for photosynthesis. The presence of satRNA in PSV-G-infected plants caused different trends in expression of DEGs associated with phosphorylation, ATP binding, and plasma membrane.

## 1. Introduction

Peanut stunt virus (PSV) is a pathogen of plants, especially legumes, which belongs to genus *Cucumovirus* [1,2,3,4,5]. The PSV genome consists of three (+)single-stranded RNAs and five open reading frames (ORFs) encoding the 1a and 2a components of the viral replicase, the movement protein, the coat protein (CP), and the 2b protein known as a viral silencing suppressor [6]. The coat and 2b proteins are synthesized from subgenomic (sg) RNA4 and sgRNA4a, respectively [7,8]. Some strains of PSV associate with satellite RNAs (satRNAs) [9,10,11]. PSV-G strain belongs to the PSV subgroup I and can infect a wide range of plants, causing severe mosaics and leaf malformations in many of them. PSV-G naturally is satRNA free. Previous analyzes have shown that PSV-G can support satRNA replication, can easily acquire satRNA from neighboring plants infected by satRNA-associated PSV-strains (e.g., PSV-P), and that satRNA addition exacerbates PSV-G symptoms in *Nicotiana benthamiana plants* [12,13].

SatRNAs are subviral pathogenic elements dependent on their respective helper viruses to provide the machinery necessary for replication and movement [14]. SatRNAs can be divided generally into two groups: small satRNAs, considered to be non-coding satRNAs, and long satRNAs, which potentially encode a single small protein that contributes functions for satRNA replication or movement. Small satRNAs are associated with viruses in genera *Cucumovirus*, *Tombusvirus*, *Sobemovirus*, *Nepovirus*, and *Polerovirus,* and many of them influence plant–virus interactions at the cellular and whole plant levels [15]. At the cellular level satRNAs alter the genetic program engaged in basic metabolism, plant defense, and certain pathways important for plant development [16,17,18,19,20]. At the whole plant level, satRNAs change virus titer and alter disease symptoms [19].

Interestingly, it was shown that the same virus associated with two types of satRNAs could cause different plant responses [21,22,23], and different virus strains, upon addition of the same satRNA, may have different pathogenesis progress. The latter case was found to occur in PSV-induced pathogenesis in *N. benthamiana* plants. It was shown that plants infected with PSV-P, which was naturally associated with satRNA, react differently to the satRNA presence under various temperature conditions, delaying or exacerbating and accelerating symptoms of disease [24]. On the other hand, addition of the same satRNA to PSV-G strain under all temperature conditions accelerated and enhanced symptoms on *N. benthamiana* plants.

The transcriptome changes associated with diseases involving non-coding satRNAs has scarcely been described for any virus. Therefore, we performed transcriptome profiling of plants inoculated with PSV-G and co-inoculated with PSV-G + satRNA and compared them to healthy (mock-inoculated) plants. In both treatments, transcripts encoding proteins in various metabolic pathways were affected during viral infection of the plant. Among these processes, the altered levels of transcripts involved in photosynthesis, carbon metabolism, RNA transport, plant–pathogen interactions, ribosome structure are reported.

## 2. Materials and Methods

### 2.1. Plant Materials and Generation of Infectious PSV-G Transcripts

*N. benthamiana* plants were rub-inoculated with PSV-G and PSV-G + satRNA-P infectious copies (in vitro synthesized transcripts) in 0.5 M phosphate, pH 7.5, after carborundum dusting. Mock-inoculated plants dusted with carborundum and inoculated with 0.5 M phosphate, pH 7.5, were used as the controls. Plants infected with PSV-G (5 μg) and infected with PSV-G + satRNA-P (5 μg of PSV-G + 500 ng of satRNA) were grown in separate chambers under 14-h light/10-h dark at 21 °C day/16 °C night and harvested when first symptoms of infection occurred (20 dpi).

Viral RNA was extracted from previously isolated viral particles of PSV-G as described in [9]. The procedures used to synthetize cDNA, which serves as template for subsequent steps, and to generate infectious copies of PSV (capped genomic transcripts with T7 promoter) were described previously [13,19]. Sequences of primers are provided in Supplementary Table S1. To generate satRNA-P transcripts, plasmid containing satRNA-P sequence was prepared as described in [13] using primers provided in Supplementary Table S1. It served as template for PCR amplification, followed by DNA extraction, and 1 μg of PCR product served as a template for an in vitro transcription reaction using a RNAMaxx High Yield Transcription Kit (Agilent Technologies, Santa Clara, CA, USA). After the reaction, 2 U of Turbo DNase (Thermo Fisher Scientific, Waltham, MA, USA) was added for DNA template digestion for 15 min in 37 °C. The resulting RNA (uncapped) was confirmed on a 1% denaturing agarose gel, followed by extraction with lithium chloride. Equimolar amounts of RNA 1, RNA 2, and RNA3 were combined for PSV inoculum.

### 2.2. RNA Extraction and Detection of Viral and satRNA in the Plants

To detect the virus RNA and satRNA, total RNA (2 μg) isolated from the upper parts was reverse transcribed using RevertAid Reverse Transcriptase (Thermo Fisher Scientific) with a random hexamer primer (Thermo Fisher Scientific) according to the manufacturer’s instructions. One µL of cDNA of postreaction mixture was used as a template in a PCR reaction (10 µL) that consisted of 1× AllegroTaq reaction buffer with 25 mM Mg^2+^ (Novazym, Poznań, Poland), 0.5 µM forward and reverse primers for detection of RNA for PSV coat protein and satRNA (Supplementary Table S1), 200 µM dNTPs, and 0.5 U AllegroTaq DNA polymerase (Novazym, Poznań, Poland). The PCRs were carried out using the MasterCycler Personal (Eppendorf, Hamburg, Germany) with an initial denaturation step at 95 °C for 3 min, followed by 35 cycles of 95 °C for 30 s, an annealing step for 25 s at 45 °C, and 72 °C for 30–55 s (depending on the anticipated product length). The reaction was completed with a final elongation step at 72 °C for 5 min.

### 2.3. Assessment of Virus Accumulation in the Plants and Validation of the Gene Expression for Chosen Transcripts by RT-qPCR

To assess virus and satRNA accumulation, two leaves of *N. benthamiana* plants were inoculated with PSV-G, with or without 500 ng of satRNA-P as described above. Upper non-inoculated leaves were harvested at 4 dpi, 11 dpi, 15 dpi, and 21 dpi. Total RNA was extracted and cDNA was synthesized. The real-time quantitative PCRs were conducted to detect PSV-G RNA 1, RNA 2, RNA 3, and satRNA using a LightCycler 480 (Roche, Basel, Switzerland). Reaction mixtures included iTaq™ Universal SYBR Green Supermix and 0.5 µM forward and reverse primers (Supplementary Table S1). The PCR program consisted of an initial denaturation step at 95 °C for 3 min, followed by 40 cycles of 95 °C for 20 s, and annealing and elongation steps at 60 °C for 1 min. Dissociation curves were generated during temperature ramping from 65 °C to 95 °C.

The gene expression levels were normalized to the level of transcription of elongation factor 1 alpha (EF1α (primers in Supplementary Table S1) which was found to be useful as a reference in previous plant-virus interaction studies [25,26].

To validate expression of chosen transcripts, cDNA was synthesized with a Maxima First Strand cDNA Synthesis Kit for RT-qPCR (Thermo Fisher Scientific) using one µg of pooled RNA samples according to the manufacturer’s instruction. RT-qPCR was carried out using the primers listed in Supplementary Table S2, three technical replicates, and standard conditions as described above. Tested genes were normalized to *EF1a* and *β-actin* gene expression with NbEF1aF/R and NbActA/2 primers (Supplementary Table S1). Standard curve for each transcript was made based on n-fold dilutions of cDNA. The reaction results were analyzed using the Relative Expression Software Tool V.2.0.13 (Qiagen, Hilden, Germany) [27].

### 2.4. Transcriptome Profiling with Species-Specific Microarrays

Total RNA was extracted from the upper parts of plants with RNeasy Plant Mini Kit (Qiagen) and labeled with Quick Amp Labeling Kit (Agilent), as previously described [28]. For each tested treatment (control plants, PSV-G infected plants and PSV-G + satRNA co-infected plants), three biological replicates were prepared. Cy5-labeled RNA was hybridized to custom Nb-105k Agilent microarrays (ID 066813) in A2 × 105K hybridization chambers (Tecan, Männedorf, Switzerland) on a HS 4800 Pro (Tecan, Männedorf, Switzerland) automatic station in a dual color hybridization mode [28]. A common reference model was used for the experiment design, where the common reference RNA (labeled with Cy3) was prepared from pooled *N. benthamiana* samples, including roots, leaves, and stems, from 4-week-old healthy plants, 10-week-old healthy plants, 6-week-old plants infected with PSV, 6-week-old plants infected with tomato torrado virus, 6-week-old plants wounded mechanically, and 6-week-old plants wounded by whitefly *Trialeurodes vaporariorum*. The intensity data for both channels were collected with a 4200AL GenePix scanner (Molecular Devices, San Jose, CA, USA).

### 2.5. Data Acquisition and Statistical Analysis

The microarray intensity data was processed with GenePix Pro 6.1 software (Molecular Devices, San Jose, CA, USA) using the morphological opening background method. The data was then analyzed through a standard analysis pipeline implemented in the R/Bioconductor limma package [29] involving the following steps: background correction (“subtract”), normalization (“loess” within array and “Aquantile” between array), quality assessment (“MA-plot” and “density plot”, spike-in analysis), and differential analysis (Bayesian linear model). Differentially expressed genes (DEGs) were identified (represented by the transcript models used for the microarray design, see [28]) with at least a two-fold expression change and a *p*-value < 0.05 (after applying Benjamini and Hochberg’s method to control the false discovery rate). Raw and normalized gene expression data from this experiment are accessible in the NCBI GEO repository via the GEO Series accession number GSE104026 [30]. Principal component analysis (PCA) was used to illustrate the relationships between the samples and replicates (plants not infected, infected with PSV-G-genomic transcripts (GT), and co-infected with PSV-G-GT + satRNA). PCA is one of the most important methods of statistical analysis of all multidimensional data and often used to reduce the dimensions of the data set where the original variables are transformed into a new set which are the “principal components”.

### 2.6. Functional Annotation of Differentially Expressed Genes and Pathway Analysis

Transcripts showing at least a two-fold change and *p*-value < 0.05 between non-infected, PSV-G infected, and PSV-G + satRNA infected plants were uploaded to the Blast2GO Pro software as queries in blastx searches against the nr and InterPro databases [31,32]. Mapping, annotation, and classification into functional categories were carried out using default settings, and statistics was performed using Blast2GO Pro. Annotations were enhanced by Annex-based gene ontology (GO) augmentation [33].

To obtain an overview of the enriched GO terms of DEG’s, we conduct the enrichment analysis based on gene counts using Fisher’s exact test [33] to identify GO terms for which the adjusted *p*-value were significant at the 0.05 level. Terms with an adjusted *p*-value < 0.05 were considered significantly enriched. Next, the z-score was calculated to assess whether the Biological Process (/Molecular Function/Cellular Components) was more likely to be decreased (negative value) or increased (positive value). Z-score was calculated according to the formula:

$$z-\mathrm{score}=\frac{\mathrm{up}-\mathrm{down}}{\sqrt{\mathrm{count}}}$$

where “up” and “down” represent the number of assigned genes which were up-regulated (logFC > 0) or down-regulated (logFC < 0) in the data and “count” was the number of genes assigned to a term. The outcomes were displayed in the bubble plot using GOplot package ver. 1.0.2 [34].

To detect clusters of functionally related genes, we employed the KEGG pathway database and the KEGG Automatic Annotation Server (KAAS) [35,36]. We also used the KEGG pathway database to estimate the significance of enrichment for each pathway with the usage of the hypergeometric tests [37].

## 3. Results

### 3.1. PSV-G Induces Strong Plant Defense but Addition of satRNA Exacerbates Symptoms Expression and Enhances Transcriptional Reprogramming in Infected Plant

**Figure 1:**
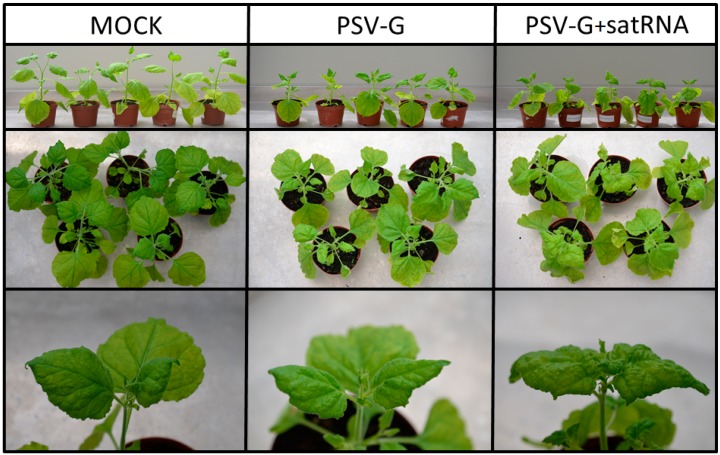
Comparison of healthy and infected *N. benthamiana* plants grown at 21 °C. Mock, not infected (left panel), infected with PSV-G-genomic transcripts (GT) (middle), and co-infected with PSV-G-GT + satRNA (right panel).

*N. benthamiana* plants were inoculated with PSV-G transcripts and symptoms were visible around 21 dpi. Symptoms included mild leaf mosaic and malformations, as well as reduced plant stature (Figure 1). Plants infected with PSV-G + satRNA showed more severe leaf mosaic and changes in leaf shape, as well as further reduction in plant stature (Figure 1). The presence of the viral RNA and satRNA by RT-qPCR was confirmed as previously described [19].

**Figure 2:**
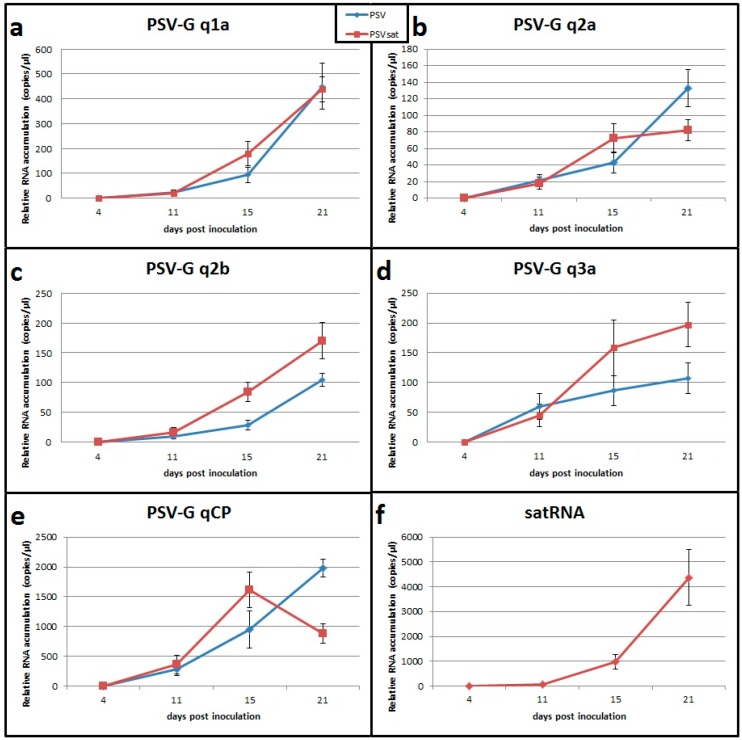
Time course analysis of PSV-G RNAs and satellite RNA (satRNA) accumulation levels in *N. benthamiana* plants. The plants were infected with PSV-G (blue) or co-infected with PSV-G + satRNA (red). (**a**) RNA encoding 1a protein; (**b**) 2a protein; (**c**) 2b protein (RNA silencing suppressor); (**d**) 3a protein (movement protein); (**e**) coat protein (CP); and (**f**) satRNA.

The levels of accumulation of the virus RNA and satRNA was monitored at 4 dpi, 11 dpi, 15 dpi, and 21 dpi. The time course analysis of the plants infected with PSV-G or PSV-G + satRNA revealed that all viral RNAs were more abundant at 15 dpi in the presence of satRNA (Figure 2a–e) (*p*-values 0.038, 0.018, 0.0, 0.007,and 0.004 for RNAs encoding PSV-1a, PSV-2a, ORF2b, movement protein (3a), and coat protein (CP), respectively). These values changed at 21 dpi. PSV-G RNA1 accumulation were similar in the presence or absence of satRNA (*p*-value 0.804) (Figure 2a). Accumulation of RNA2 reached a plateau between 15–21 dpi in the presence of satRNA (*p*-value 0.195), whereas PSV RNA2 continued to increase in the absence of satellite (*p*-value 0.0). Analysis with primers specific to RNA encoding ORF2b (reflecting results for both genomic RNA2 and sgRN4A) showed that level of viral RNA was higher in PSV-G + satRNA-infected plants at 15 and 21 dpi (*p*-value 0.0 and 0.001, respectively). The viral RNAs encoding the 2b and the 3a were more abundant (*p*-value 0.001 and 0.003, respectively) and the viral RNAs encoding PSV-2a and the CP (the latter one reflects the level of both genomic RNA3 and sgRNA4) were less abundant (*p*-value 0.0 and 0.0, respectively). SatRNA accumulation was low until 11 dpi, then rapidly increased (Figure 2f).

### 3.2. Functional Categorization of PSV and satRNA Responsive Genes Indicates Deep Changes in Primary Metabolism and Stress Response in Infected Plants

Species-specific microarrays (Nb-105k) were used to evaluate the transcriptomic responses of *N. benthamiana* plants to PSV-G or PSV-G + satRNA infection. The microarrays were prepared using Agilent in situ oligonucleotide synthesis technology and represent transcripts from two recent de novo assemblies of *N. benthamiana* RNA-Seq data [38,39].

**Figure 3:**
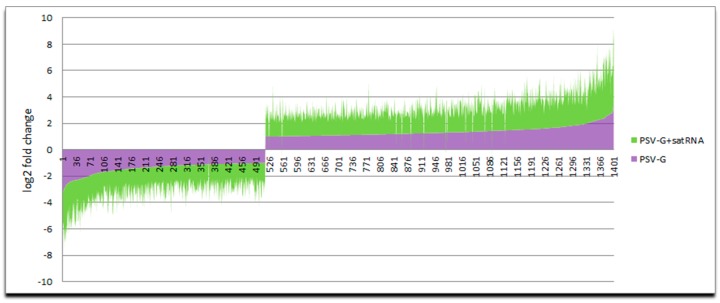
Levels of differentially expressed genes (DEGs) expressed in common by both PSV-G and PSV-G + satRNA are shown.

The Principal Component Analysis (PCA) was used to illustrate the relationship between group of samples to show the correlation between the samples (Supplementary Figure S1). Transcriptome profiling detected 1751 statistically significant DEGs (*p* < 0.05), with at least 2-fold change when PSV-G infected plants were compared with non-infected control plants and 9379 DEGs when PSV-G + satRNA-infected were compared with non-infected plants. Comparison of the common DEGs between PSV-G- and PSV-G + satRNA-infected plants showed that the fold changes were usually higher in the PSV-G + satRNA-infected plants (Figure 3). These results show that there was a much stronger plant transcriptome response to PSV-G + satRNA infection compared with PSV-G infection (Figure 3). Compared DEGs were identified using Benjamini and Hochbert’s method (*p*-value < 0.05).

**Figure 4:**
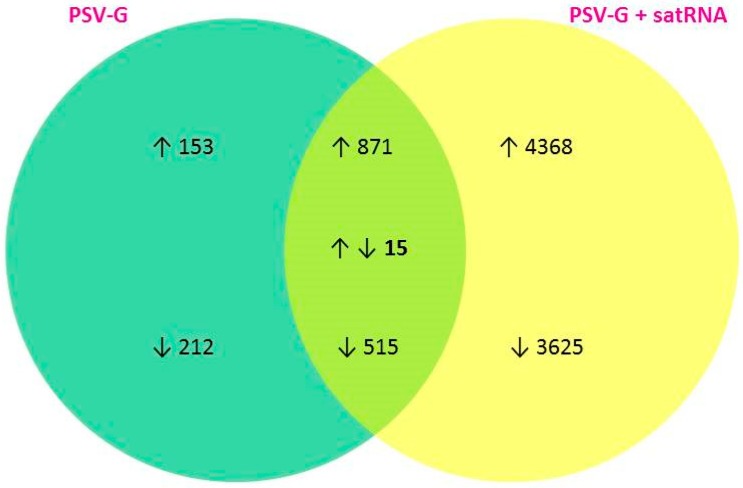
Venn diagram showing unique and common DEGs in PSV-G and PSV-G + satRNA plants. The arrows indicate up-regulated (↑) and down-regulated (↓) differentially expressed genes (DEGs).

All the DEGs identified in PSV-G and PSV-G + satRNA infected plants are listed in Supplementary Table S3. The numbers of common and unique DEGs identified in PSV-G- and PSV-G + satRNA-infected plants were visualized in a Venn diagram (Figure 4).

### 3.3. Up-Regulated and Down-Regulated Plant Transcripts upon PSV Infection or PSV + satRNA Co-Infection

**Figure 5:**
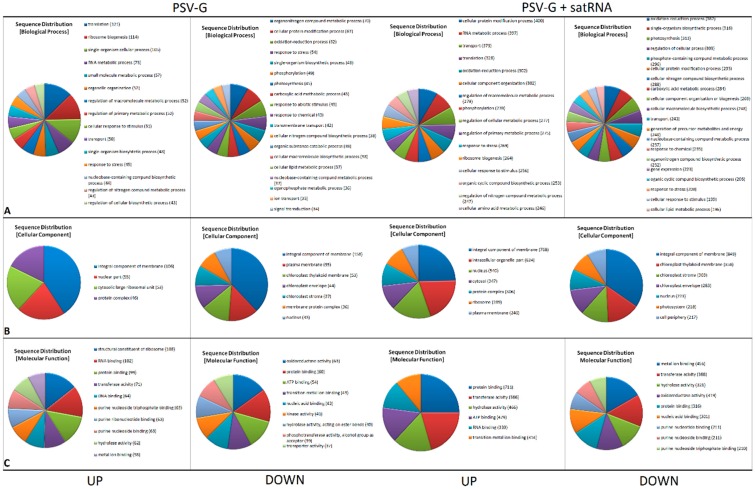
Gene ontology (GO) terms for differentially expressed genes (DEGs) of *Nicotiana benthamiana* infected with PSV or PSV + satRNA presented as multi-level pie charts. The distribution of GO terms was analyzed separately for up-regulated and down-regulated DEGs. (**A**) Biological Process; (**B**) Cellular Component; and (**C**) Molecular Function.

Plant inoculation with PSV-G resulted in 1029 up-regulated and 722 down-regulated DEGs and inoculation with PSV-G + satRNA resulted in 5239 up-regulated and 4140 down-regulated DEGs, identified at the time of plant harvest. The total numbers of up-regulated and down-regulated transcripts, and numbers of DEGs with blast hits and Blast2Go annotations are shown in Table 1, and further details are in Supplementary Table S3. The DEGs were assigned under the three main GO categories: Biological Process, Cellular Compartment, and Molecular Function (Figure 5).

The up-regulated DEGs in both PSV-G and PSV-G + satRNA-infected plants in both treatments were associated with translation, ribosome biogenesis, RNA metabolic process, response to stress, and transport (Biological Process). Importantly, in the plants co-infected with PSV-G + satRNA, the most abundant up-regulated transcripts were associated with cellular protein modification process (400 DEGs), including phosphorylation (278 DEGs). Also, transcripts related to oxidation-reduction process and cellular component organization were highly represented (302 DEGs for both terms) (Figure 5A). The most abundant up-regulated DEGs were associated with integral components of the membrane (for both treatments), nucleus, cytosolic large ribosomal subunit and protein complexes (for PSV-G treatment), and with plasma membrane and chloroplast structures (especially for PSV-G + satRNA treatment) (Cellular Components) (Figure 5B). The prevailing up-regulated DEGs were associated with structural constituent of ribosome (especially in PSV-G-infected plants), transferase and hydrolase activity, and with binding to RNA, proteins, and metal ions activity (for both treatments) (Molecular Function) (Figure 5C). Additionally, in PSV-G + satRNA-co-infected plants DEGs associated with ATP binding are among the most abundant.

Among the biological processes that were down-regulated in PSV-G- and PSV-G + satRNA-infected plants, DEGs associated with oxidation-reduction process, photosynthesis, transport, nitrogen compound biosynthetic process, cellular lipid metabolism process were highly abundant (Figure 5A). Down-regulated DEGs were primarily associated with membranes, chloroplasts and the nucleus (Figure 5B). Importantly, the number of down-regulated DEGs in plants co-infected with PSV-G + satRNA related to photosystem term was very high (218 transcripts). For PSV-G the downregulated DEGs were attributed among others to oxidoreductase activity, ATP binding, and kinase activity. For PSV-G + satRNA the downregulated DEGs were among others attributed to metal ion binding and transferase and hydrolase activities (Figure 5C). Prevailing GO terms among commonly up-regulated or down-regulated DEGs in plants infected by PSV-G and PSV-G + satRNA were listed in Supplementary Table S4.

**Figure 6:**
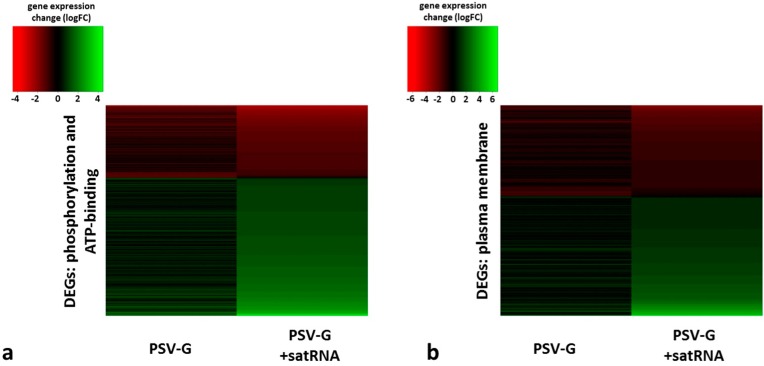
Heatmaps showing distribution of GO terms assigned to the DEGs of PSV-G and PSV-G + satRNA infected plants. (**a**) Combined terms for phosphorylation (Biological Process) and ATP binding (Molecular Function); (**b**) plasma membrane (Cellular Component) in PSV-G (left) and PSV-G + satRNA (right) infected plants.

The results showed that the presence or absence of satRNA in the PSV-G infected plants had opposite effects on some of the most abundant GO terms under the three functional categories, Biological Process, Cellular Component, and Molecular Function, as shown in Table 2. In both PSV-G- and PSV-G + satRNA-infected plants, the up-regulated DEGs assigned to Cellular Component terms were abundant in the nucleus, integral components of membranes, and protein complexes. Interestingly, a larger number of the up-regulated DEGs were assigned to the plasma membrane in the PSV-G + satRNA infected plants than it was observed in down-regulated pool of DEGs related to this term for PSV-G + satRNA-infected plants. Contrary, in the PSV-G-infected plants, a larger number of DEGs related to plasma membrane were down-regulated (Figure 6) than up-regulated. For both treatments, a large number downregulated DEGs were associated with various chloroplast parts (membrane, envelope, stroma, thylakoids). Additionally, in the PSV-G + satRNA-infected plants, a large group of down-regulated DEGs were associated with the photosystem and cell periphery.

Similar situation as described above was observed for transcripts associated with phosphorylation, where both, up-regulated and down-regulated DEGs were found in both PSV-G- and PSV-G + satRNA-infected plants. However, in PSV-G-infected plants, transcripts involved in phosphorylation were among the highly represented down-regulated DEGs (constituting more than 5% of all assigned GO terms), whereas in PSV-G + satRNA infected plants among up-regulated ones (Table 2, Figure 6a). Consistently, in the PSV-G + satRNA-infected plants, the DEGs associated with ATP binding were rather up-regulated, whereas, in the PSV-G-infected plants, the higher number of DEGs associated with ATP binding were down-regulated (Table 2).

**Figure 7:**
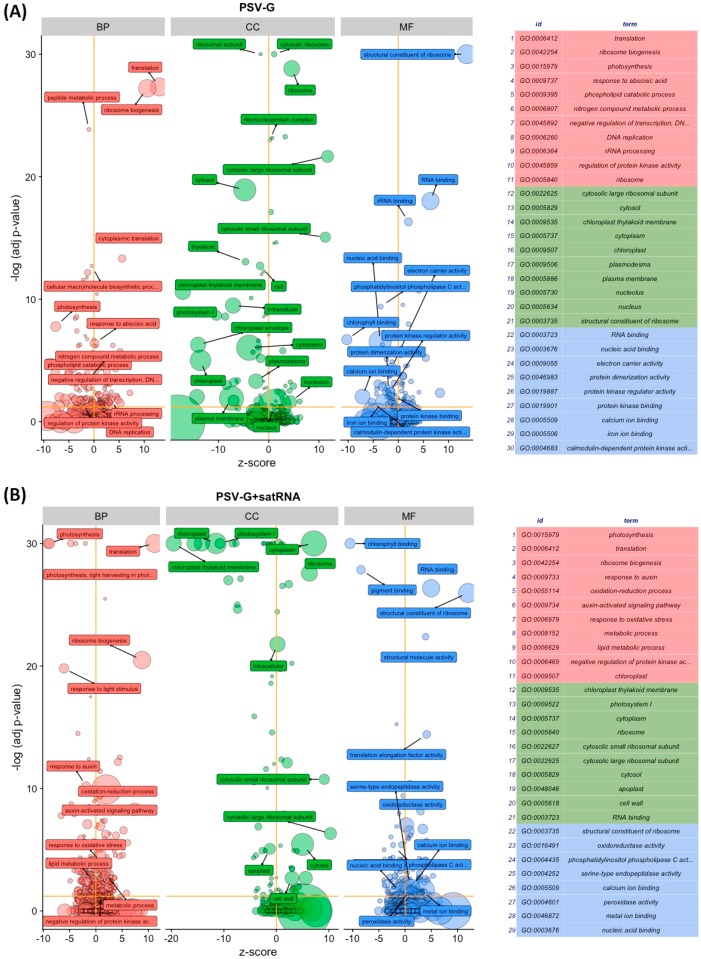
Gene ontology terms for DEGs of *Nicotiana benthamiana* infected with PSV-G (**A**) and PSV + satRNA (**B**). The z-score was assigned to the x-axis and the negative logarithm of the adjusted *p*-value to the y-axis (the higher the more significant). The area of the displayed circles is proportional to the number of genes assigned to the term and the color corresponds to the three categories: Biological Process (BP, red), Cellular Component (CC, green) and Molecular Function (MF, blue). The circles were labeled with the GO term name. On the right side a table connecting the IDs and terms is displayed. Only some of significant circles were labelled due to the limited space and the overlap of the circles. A threshold for the labeling was set based on the negative logarithm of the adjusted *p*-value (>1.3 which gives only GO terms that are significant at the 0.05 level).

The bubble plots presented in Figure 7 gave an overview of the enriched terms for plants infected with PSV-G (Figure 7A) and PSV-G + satRNA (Figure 7B).

### 3.4. PSV-Infected Plants Show Down-Regulation of Genes Involved in Photosynthesis and Up-Regulation of Genes Associated with Ribosome

Pathway analysis was performed using KAAS, KEGG pathway database, and then hypergeometric tests was performed to estimate the significance of enrichment for each pathway, as described in M&M. Up-regulated and down-regulated DEGs in the PSV-G- and PSV-G + satRNA-infected plants were analyzed separately. The analysis of the top 15 most affected pathways (Supplementary Table S5) showed that following each treatment, metabolic pathway and biosynthesis of secondary metabolites were affected, and both up- and down-regulated DEGs were involved.

**Figure 8:**
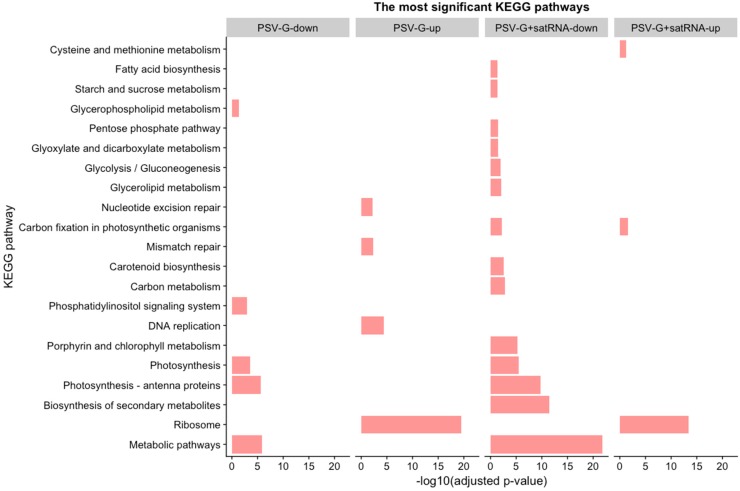
A subset of affected KEGG pathways involving up-regulated and down-regulated DEGs in plants infected with PSV or PSV + satRNA. The significant KEGG pathway terms for up- and down- differentially regulated genes were assigned to the y-axis and the negative logarithm of the adjusted *p*-value to the x-axis (the longer the more significant). Only the significant terms are shown and the bars are ordered based on their significance (seen from below).

The metabolic pathways associated with ribosome and photosynthesis were among the most statistically significant (*p* < 0.05) (Figure 8). The DEGs engaged in in ribosome pathway were up-regulated while in photosynthesis and antenna proteins were down-regulated in the PSV-G- and PSV-G + satRNA-infected plants.

**Figure 9:**
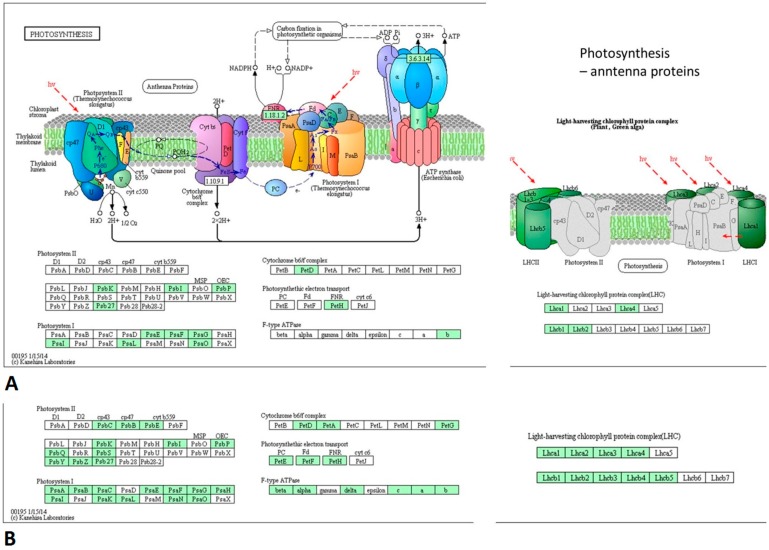
Down-regulated DEGs (marked in green) associated with photosynthesis (left) and antenna proteins (right). (**A**) PSV-G infected plants; (**B**) PSV-G + satRNA-co-infected plants. Visualization was obtained from KAAS [35].

The differently expressed transcripts related to photosynthesis and antenna proteins were shown in Figure 9. Results showed that in PSV-G and PSV-G + satRNA-infected plants, this pathway and structures were affected. However, the number of down-regulated transcripts was much higher in the presence of symptom exacerbating satRNA. Among the affected transcripts were these associated with proteins constituting photosystem I and II and light-harvesting chlorophyll protein complex (LHC).

### 3.5. Processes Associated with Plant–Pathogen Interaction and Plant Defense

**Figure 10:**
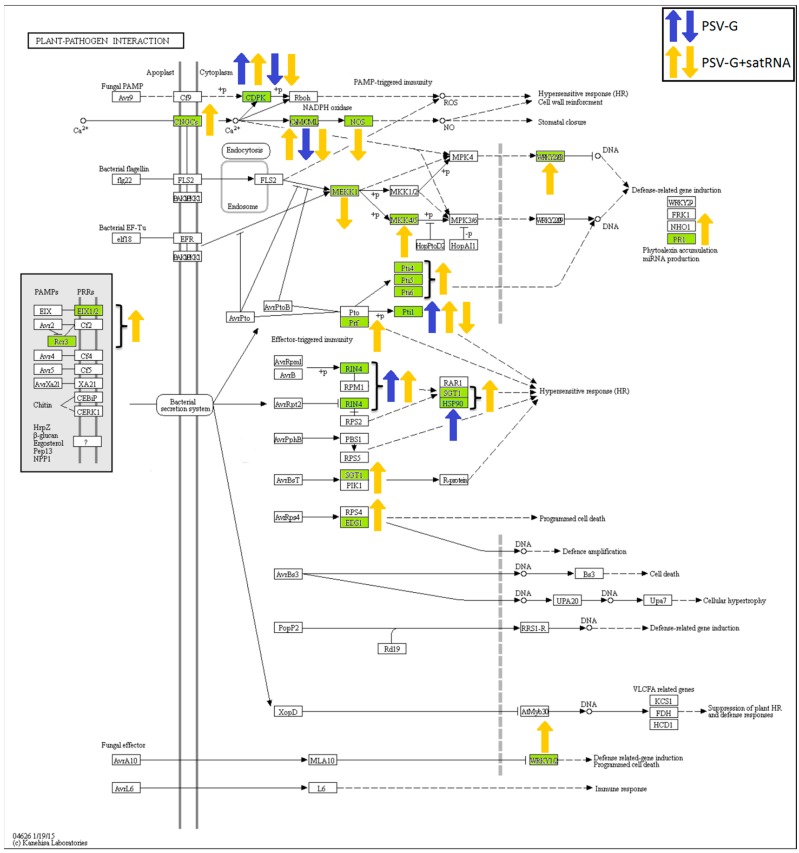
Differentially expressed genes (green) associated with plant–pathogen interaction in PSV and PSV + satRNA infected plants. Up- and down-regulated DEGs are marked with arrows. The results are visualized in KAAS [35].

Many up-regulated DEGs associated with plant–pathogen interaction were detected in the PSV-G- and PSV-G + satRNA-infected plants, including genes associated with hypersensitive response. In the PSV-G + satRNA infected plants, genes associated with defense-related gene induction (PR1 synthesis) and programmed cell death were also detected (Figure 10). The down-regulated DEGs associated with plant–pathogen interaction were associated with only a few pathways leading to stomatal closure and cell wall reinforcement (both treatments).

**Figure 11:**
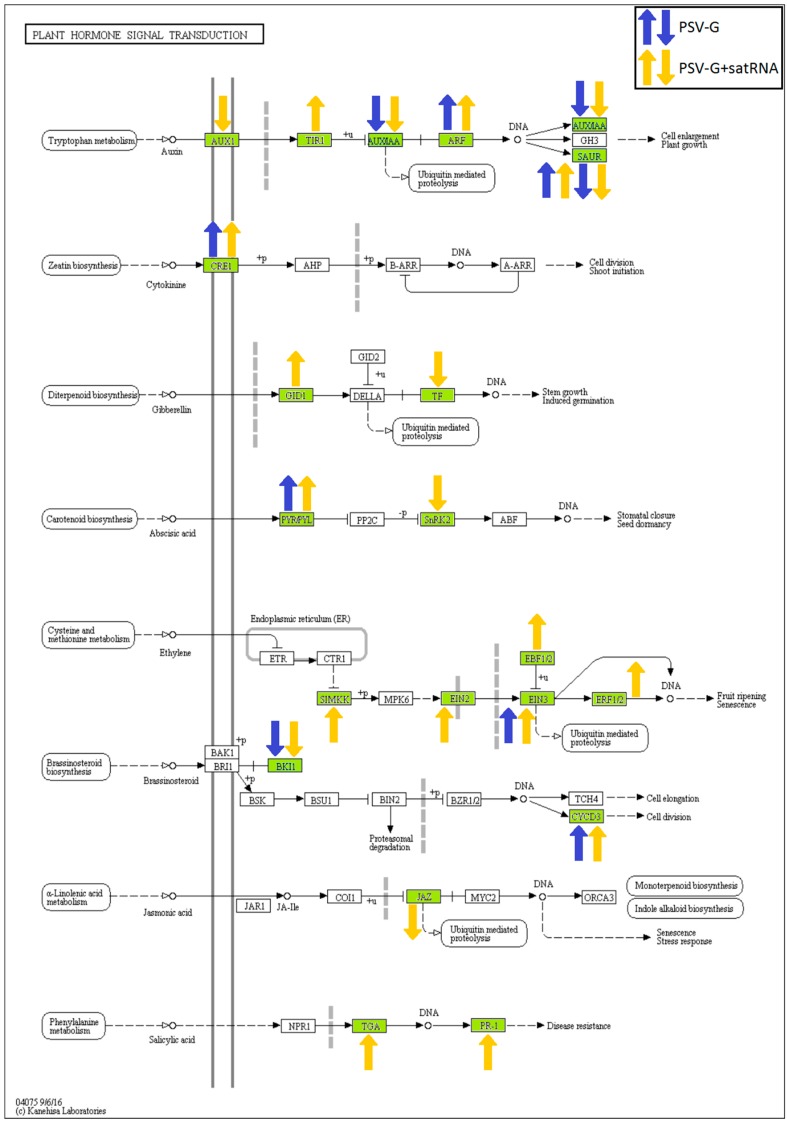
Differentially expressed genes (green boxes) associated with hormone signaling pathways in PSV and PSV + satRNA-infected plants. Up- and down-regulated DEGs are marked with arrows. The results are visualized in KAAS [35].

Plant hormonal signaling pathways were also affected in the PSV-G and PSV-G + satRNA-infected plants (Figure 11). Detailed analysis showed the presence DEGs encoding proteins involved in auxin and brassinosteroid signaling. Among the up-regulated DEGs, pathway associated with ethylene signal transduction were represented, especially in the PSV-G + satRNA infected plants. On the other hand, up-regulation of transcripts associated with salicylic acid signaling was observed only in plants infected with PSV-G + satRNA (Figure 11). Noticeably, for some genes encoding proteins involved in plant hormonal signaling and plant–pathogen interaction, both up- and down-regulation of the same transcripts were observed for the same treatment condition; for example, CDPK (calcium-dependent protein kinase) and SAUR (small auxin up RNAs) (Figure 10 and Figure 11, respectively). This contradiction may be explained by the differential regulation of various isoforms of these proteins or the probe used in the microarray may be common for a number of transcripts with highly similar sequences. It is also possible that some parts of the assemblies of *N. benthamiana* RNA-Seq data (v3) used for the microarray probe design may be chimeric, leading to inconsistency between the gene annotation and the reported gene expression [28].

### 3.6. Posttranscriptional Gene Silencing (PTGS)

Some of the DEGs were predicted to encode proteins that participate in PTGS in PSV and PSV + satRNA infected plants. In the PSV-G + satRNA infected plants, DEGs encoding Ago1, 2, 5, RNA-dependent RNA polymerases 1 and 6 (RDR1, 6), and Dicer-like ribunucleases 1 and 4 (DCL1, 4) were up-regulated, whereas in PSV-G infected plants, DEGs encoding Ago1, 2, RDR1, and DCL1, 4 were up-regulated. In both treatments, the level of Ago2 was particularly high (Table 3).

### 3.7. Validation of Transcriptomic Data by RT-qPCR

We selected 11 DEGs (7 up-regulated and 4 down-regulated) that showed statistically significant changes (at least 2-fold) in the transcriptomic profiling of both the PSV-G and PSV-G + satRNA infected plants (Supplementary Table S3) for validation by RT-qPCR. The direction of observed change (increase/decrease) was confirmed for all genes (Table 4 and Table 5), although the result for *PR2* was not statistically significant for the PSV-G-infected plants (*p*-value 0.46) (Table 4). Expression level changes are usually smaller in RT-qPCR analysis compared with those determined from microarray data. This result may reflect the fact that the RNA for transcriptome profiling was used shortly after plant harvesting and RNA isolation, whereas, for the RT-qPCR analysis, the same material was stored at −80 °C until the transcriptome was sequenced and the follow-up analysis was finished before the DEGs were selected.

## 4. Discussion

The symptoms of infection by many plant viruses are attenuated in infected plants when the viruses are associated with satRNAs [18,40,41,42,43]. However, strong exacerbation of symptoms has also been described, including those for cucumoviruses associated with satRNAs [16]. The impact of satRNAs associated with some strains of PSV has been reported to from attenuate the severity of pathogenesis severity in the case of satG and satWC of PSV [10,11], to have no visible effect on pathogenesis in the case of satV of PSV [11]. This study demonstrates that the naturally satRNA-free PSV-G strain easily acquired satRNA and such co-infection strongly exacerbated and accelerated diseases symptoms in infected plants. This study reports the first to our knowledge analysis of the whole transcriptome changes that are associated with symptom exacerbation by satRNA in virus infected plants.

Analyses of the accumulation of viral RNAs revealed that the more severe symptoms observed on plants co-infected with PSV-G + satRNA may be associated with faster replication of the virus and a stronger plant response. This idea was supported by the considerably higher number and higher fold change of DEGs found in *N. benthamiana* co-infected with PSV-G + satRNA compared with PSV-G infected plants. Interestingly, the KEGG analysis indicated that most of the DEGs observed in PSV-G and PSV-G + satRNA-infected plants were involved in metabolic pathways, biosynthesis of secondary metabolites, which is similar to reports involving the capsicum chlorosis virus–capsicum interaction [44]. GO analysis revealed that among the up-regulated DEGs in the PSV-G and PSV-G + satRNA infected plants, a large number were associated with translation, ribosome biogenesis, and RNA metabolic processes, and this was more pronounced in the PSV-G + satRNA infected plants (Figure 7). These results indicated huge reprogramming of the cell into synthesizing machinery may have occurred, possibly as a result of both viral component synthesis and induced plant response. Induction of gene expression for synthesis of constituents that participate in translation and ribosome structure/biogenesis has also been found in other plant–virus interactions, including in bean common mosaic virus infection of common bean (*Phaseolus vulgaris* L.) and beet necrotic yellow vein virus interaction with *N. benthamiana* [45,46].

The pathways most influenced by virus infection were metabolic pathways, ribosome and photosynthesis. Genes involved in photosynthesis were down-regulated in both PSV-G and PSV-G + satRNA infected plants, but in the latter case the number of DEGs and their fold-change were much bigger. Similar observations have been reported in numerous plant–virus pathosystems [19,24,47,48,49], which suggests that this is a relatively common phenomenon and that cellular photosynthetic components participate in plant immunity signaling [50]. This finding is directly associated with another observation; that is, the cellular component severely affected during PSV infection was chloroplast, involving DEGs that encode proteins that are components of the chloroplast thylakoid membrane, chloroplast envelope, stroma, and photosystem. KEGG analysis confirmed this finding and also indicated the down-regulation of genes encoding antenna components. DEGs that were down-regulated in PSV infected plants were strongly magnified in PSV-G + satRNA infected plants, which also was reflected by more severe mosaics on these plants. This is additional evidence that chloroplasts, photosystems, and antenna complexes [e.g., light-harvesting complex II (LHCII)] and their associated components play crucial roles in virus-induced diseases and in plant immunity signaling [50,51,52,53].

Phosphorylation is important in early signaling events during pathogen infection [54,55]. In this study, we did our analyses when the first symptom of infection was observed and the viral RNA level was already high in plant cells; therefore, our results cannot reflect the early stages of infection. However, the general direction of observed changes in the DEGs associated with phosphorylation and ATP binding was most interesting. Numerous up- and down-regulated DEGs from these categories were detected in both PSV-G and PSV-G + satRNA infected plants. However, in the PSV-G infected plants, more of the DEGs associated with ATP binding and phosphorylation were found to be down-regulated compared with in PSV-G + satRNA infected plants where the number of up-regulated DEGs associated with these categories was considerably higher than the number of down-regulated DEGs. This result indicates that phosphorylation may be one of the driving forces in immune signaling during disease exacerbating satRNA co-infection.

KEGG analysis of pathways indicated that DEGs associated with processes leading to hypersensitive response and plant defense-related gene induction, including *PR1,* were up-regulated in both treatments, but more DEGs were found for the PSV-G + satRNA infected plants. Pathogenesis-related proteins are frequently accumulated upon pathogen attack, and they have been found in other plant–virus transcriptome analyses. *PR1* overexpression has been observed in many virus-infected plants and constituted an important constituent of the plant stress response [44]. Induction of *PR1* expression is a landmark and marker gene of salicylic acid-associated pathway activation leading to acquired resistance in plants [56,57]. Analysis of DEGs involved in hormone-mediated signaling pathways upon PSV infection showed that the salicylic acid pathway was involved in plant response to PSV-G + satRNA co-infection. However, DEGs associated with the ethylene pathway showed the biggest response, with one up-regulated gene (*EIN3*) detected in PSV-G infected plants and five up-regulated genes detected in PSV-G + satRNA infected plants. DEGs (from 1 to 2) encoding proteins associated with the cytokine, gibberellin, abscisic acid, and brassinosteroid pathways were found in both treatments. Considerable perturbation (with genes up- and down-regulated) were also found in the auxin signaling pathway. Components involved in ethylene signaling have also been found to be altered upon infection with other viruses, including CMV, tomato spotted wilt virus, and potato virus X [58]. Other phytohormones and associated pathways were also proven to play important roles in defense against pathogens, with auxins having a modulatory effect on the plant defense signaling system, and crosstalk with other phytohormones, including cytokines, in the processes of growth and immunity [59]. Tobacco mosaic virus was reported to reprogram auxin transcriptional responses enhancing virus phloem loading [60]. These results indicate induction of plant resistance and engagement of cellular signaling pathways occur in both virus transmission inside a host plant and induction of plant resistance responses upon PSV + satRNA infection.

Analysis of GO terms assigned to the DEGs showed that many of them were involved in response to stress and response to stimuli. However, the numbers of up-regulated and down-regulated DEGs assigned to these categories were comparable for both treatments which implies that very dynamic processes occur in infected plant cells. In our previous proteomic analysis of disease progress delayed in *N. benthamiana* by co-infection with satRNA and PSV-P, it was observed that most differentially regulated stress-related proteins were less abundant [19]. This difference between PSV-G + satRNA and PSV-P + satRNA infection was probably caused by the exacerbating effect of satRNA in PSV-G coinfection and thus the more pronounced plant response was reflected by the higher number of up-regulated stress-related genes.

Some of the up-regulated DEGs were involved in PTGS, the major defense mechanism employed by plants to target RNA viruses. Among the transcripts involved in PTGS, PSV-G infection led to up-regulation of *AGO1, AGO2, RDR1, DCL1*, and *DCL4*, although the latter three were only slightly up-regulated. Co-infection with PSV-G + satRNA caused higher up-regulation of aforementioned genes and additionally up-regulation of *AGO5* and *RDR6.* The *AGO2* expression level was the highest among them; 4-fold change in PSV-G infected plants and almost a 10-fold change in PSV-G + satRNA infected plants. This result is similar to that reported for another *N. benthamiana*—virus pathosystem in which *AGO2* was found to be directly associated with anti-tomato bushy stunt virus RNA silencing, and thus supposed to play an important role in antiviral defense [61]. In *Arabidopsis* infected by turnip crinkle virus and in another cucumovirus–cucumber mosaic virus (CMV), strong antiviral defense of AGO2 against these viruses has been reported [62]. AGO1 is considered to be next to AGO2 as a major antiviral AGO that acts against RNA viruses in *Arabidopsis* [63], but AGO1 was also found to act in *N. benthamiana* defense against, for example, tomato ringspot virus [64]. AGO5 was reported to have a minor role in antiviral defense [63]; however, it was found to be up-regulated 2,3-fold in cassava infected with cassava brown streak virus [65]. In this study, AGO5 was up-regulated by over 3-fold. RNA-dependent RNA polymerase (RdRp) plays an important role in PTGS by catalyzing double-stranded RNA synthesis, which constitutes the template for dicer-like protein (DCL) cleavage. Here, upon PSV-G infection *RDRP1* was found to be weakly up-regulated, and this effect was enhanced by PSV-G + satRNA co-infection, and the level of *RDRP6* was also increased in the latter case. *RDR1* was found to be strongly involved in the antiviral response against many viruses including potato virus Y [66], while *RDR6* was reported to be involved in short-range RNA silencing in *N. benthamiana* [67], which may imply the signal for PTGS was better for plants co-infected with satRNA. For the *DCLs*, the very weak up-regulation of *DCL1*, which functions in endogenous miRNAs biogenesis [68], and *DCL4* in PSV-G infected plants was further enhanced in the presence of satRNA. DCL4 was found previously to restrict systemic movement of zucchini yellow mosaic virus [69].

## 5. Conclusions

An old concept in plant virology was to use satRNA to diminish the damage associated with their helper virus infection, because satRNA usually was observed as attenuating symptom expression and pathogenesis severity. Later it was found that satRNA may “escape” from its original helper virus strain and associate with the strain that supports its replication. As a result, co-infection with satRNA can have different courses and instead of attenuating symptoms, satRNA can exacerbate symptom expression.

Here, we examined the relationship between PSV-G strain and satRNA, which—although with another strain from the same subgroup of PSV (PSV-P) weakens pathogenesis—here was responsible for the acceleration and exacerbation of the disease course. Our results confirm that the same satRNA can make the disease more severe, and leads to transcriptome reprograming, altering expression of additional transcripts in PSV-G plus satRNA that is more extensive then in plants infected with PSV-G alone. Together, our results contribute to the understanding of the high complexity of plant–virus–satRNA interactions and the strong influence of various factors on disease progress and plant response.

## Figures and Tables

**Table 1:** Differentially expressed genes (DEGs) of *Nicotiana benthamiana* infected with PSV-G or PSV-G + satRNA. Percentage of DEGs with Blast hits and percentage annotated DEGs using the Blast2Go Pro server are shown.

Treatment Condition/Tendency	Number of DEGs	With Blast Hits	B2GO Annotated
PSV-G/up-regulated	1024	856 (83%)	611 (59%)
PSV-G/down-regulated	727	578 (80%)	445 (62%)
PSV-G + satRNA/up-regulated	5239	4469 (85%)	3453 (66%)
PSV-G + satRNA/down-regulated	4140	3366 (81%)	2559 (62%)

**Table 2:** Contrasting effects (↑—increase and ↓—decrease) of the absence or presence of satellite RNA (satRNA) in PSV-G infected *Nicotiana benthamiana* plants. The distribution of GO terms assigned to the differentially expressed genes under the three GO functional categories is shown.

Opposite Effects of PSV Infection in the Absence of Presence of satRNA (Number of Seqs)		
	PSV-G	PSV-G + satRNA
phosphorylation (biological process)	↓ (49)	↑ (278)
plasma membrane (compartment distribution)	↓ (55)	↑ (240)
ATP binding (molecular function)	↓ (54)	↑ (429)

**Table 3:** Expression levels of genes that encode proteins involved in posttranscriptional gene silencing.

Log2 Change in Gene Expression for PSV-G	Log2 Change in Gene Expression for PSV-G + satRNA	*p* Value for PSV-G	*p* Value for PSV-G + satRNA	Function/Sequence Name/BlastN ID
0.327	**1.246**	0.562778	**0.000858**	Protein argonaute 5 Nbv3K625761138/XM_019408655.1
**1.059**	**1.249**	**0.000215**	**1.00E−06**	Protein argonaute 1 (similar to) RC_Nbv3K685820100/XM_019369300.1
**0.946**	**1.299**	**0.007104**	**3.70E−05**	Protein argonaute 1B Nbv3K785652119/XM_016590746.1
**0.919**	**1.389**	**0.002047**	**1.00E−06**	Protein argonaute 1 (similar to) Nbv3K705831504/XM_019369300.1
**1.019**	**1.42**	**0.003157**	**8.00E−06**	Protein argonaute 1-like (similar to) RC_Nbv3K705830082/XM_019369300.1
**1.072**	**1.521**	**0.001496**	**2.00E−06**	Protein argonaute 1-like RC_Nbv3K625765725/XM_019369300.1
0.25	**1.654**	0.669212	**2.50E−05**	Protein argonaute 5 RC_Nbv3K625760429/XM_009788807.1
**1.192**	**1.736**	**0.003733**	**7.00E−06**	Protein argonaute 1A Nbv3K585682683/XM_016630483.1
**1.473**	**1.958**	**2.60E-05**	**0**	Protein argonaute 1B Nbv3K685814294/KR942296.1
**1.178**	**2.135**	**0.001783**	**1.00E−06**	Protein argonaute 1 Nbv3K665799914/XM_009771957.1
**2.195**	**3.286**	**2.60E-05**	**0**	Protein argonaute 2 (similar to) RC_Nbv3K585706870/XM_016629769.1
0.286	**1.283**	0.353833	**3.00E−06**	RNA-dependent RNA polymerase 6 (AtRDRP6) (similar to)/Nbv3K585707928/XM_019404979.1
**0.83**	**2.252**	**0.020257**	**0**	RNA-dependent RNA polymerase 1 (AtRDRP1) (similar to)/Nbv3K585714562
0.812	**2.276**	0.155813	**1.30E−05**	RNA-dependent RNA polymerase 1 (AtRDRP1) (putative) Nbv3K745620210/AY574374.1
0.421	**1.093**	0.206175	**9.40E−05**	Endoribonuclease Dicer homolog 1 (similar to) RC_Nbv3K585722110/XM_009796481.1
**0.752**	**1.314**	**0.036**	**5.30E−05**	Dicer-like protein 4 (putative) Nbv3K625768999/XM_009767633.1
**0.623**	**1.419**	**0.015192**	**0**	Endoribonuclease Dicer homolog 1 (OsDCL1) (similar to) RC_Nbv3K585722111/XM_016616158.1
0.628	**1.492**	0.099327	**2.00E−05**	Endoribonuclease Dicer homolog 4 (OsDCL4) (probable) RC_Nbv3K585724251/XM_009767634.1

Statistically significant changes are marked in bold.

**Table 4:** Validation of the gene expression in PSV-G treated plants by RT-qPCR compared to the results obtained from transcriptomics analysis (red-colored cells represent up-regulated genes, while, the blue-colored—down-regulated ones).

Gene	Results Obtained from RT-qPCR			Results Obtained from Transcriptomics Analysis		
	Expression	Std. Error	*p*-Value	Expression	Std. Error	*p*-Value
*AGO2*	2.761	0.449–16.281	0.002	4.594		
*EF1delta*	7.347	3.358–16.188	0	4.317		0
*Hsp17.3*	19.034	5.997–49.014	0	6.821		
*MBF1C*	4.089	0.901–18.471	0	5.242		0
*PNO1*	5.757	2.185–14.410	0	3.944		
*PR1*	2.178	0.607–7.063	0.006	4.347		0
*PR2*	1.222	0.337–4.482	0.46	5.502		0
*ABCC*	0.148	0.093–0.241	0	0.177		0
*IRT1*	0.07	0.008–0.656	0	0.203		0
*PAP1*	0.065	0.024–0.161	0	0.189		0
*PPCK*	0.431	0.264–0.701	0	0.308		0

**Table 5:** Validation of the gene expression in PSV-G + satRNA treated plants by RT-qPCR compared to the results obtained from transcriptomics analysis (red-colored cells represent up-regulated genes, while, the blue-colored—down-regulated ones).

Gene	Results Obtained from RT-qPCR			Results Obtained from Transcriptomics Analysis		
	Expression	Std. Error	*p*-Value	Expression	Std. Error	*p*-Value
*AGO2*	5.004	0.829–34.453	0	9.849		0
*EF1delta*	2.607	1.113–5.838	0	3.732		
*Hsp17.3*	6.151	1.968–25.740	0	10.556		
*MBF1C*	4.911	1.553–16.784	0	25.992		
*PNO1*	2.981	1.134–7.371	0	8.000		0
*PR1*	32.49	9.798–85.277	0	51.984		0
*PR2*	14.488	4.620–45.487	0	147.033		
*ABCC*	0.206	0.145–0.313	0	0.330		0
*IRT1*	0.162	0.026–1.129	0	0.287		0
*PAP1*	0.048	0.026–0.084	0	0.144		0
*PPCK*	0.268	0.198–0.408	0	0.353		0

## Supplementary Materials

The following are available online at http://www.mdpi.com/1999-4915/10/9/449/s1, Table S1: Primers used in this study to satRNA synthesis, viral RNAs detection and virus accumulation assessment; Table S2: Primers used in this study for validation the gene expression for chosen transcripts by RT-qPCR; Table S3: Differentially Expressed Genes Identified in PSV-G and PSV-G + satRNA infected plants, with information on probes used, statistical data, and gene ontology data; Table S4: Prevailing terms among commonly up-regulated or down-regulated DEGs in plants infected by PSV-G and PSV-G + satRNA; Table S5: Top 15 most affected pathways involving DEGs in PSV-G and PSV-G + satRNA infected plants; Figure S1: Principal component analysis of the tested samples in the experiment. Cases are grouped by the biological replicates (mock inoculated plants (Z), PSV-G alone (G), PSV-G and satRNA (GS)).
