# Supplementary material for: The Defense Response of Nicotiana benthamiana to Peanut Stunt Virus Infection in the Presence of Symptom Exacerbating Satellite RNA
Source: Viruses. 2018 Aug 23;10(9):449. doi: 10.3390/v10090449 (PMC6165542; doi:10.3390/v10090449)
Supplement: Supplementary file 1 [file viruses-10-00449-s001.zip › Supplementary Files/Suppl. table 2.docx]

**Table S2** Primers used in this study for validation the gene expression for chosen transcripts by RT-qPCR.

| Primer name | Primer sequence (5’-3’) | Amplicon length [bp] | Gene |
| --- | --- | --- | --- |
| NbABCC | F: GGCTGACCGATTTAATTTGC | 173 | ABC transporter C family member 5 |
|  | R: GTCACCTTTGGTTGGCAGTT |  |  |
| NbAGO2 | F: TGTTTCGGGTCGTATCCTTC | 160 | Protein argonaute 2 |
|  | R: GCGTTCCTGGGAGCTAAAAT |  |  |
| NbEF1delta | F: TGGCTGTTGCATTCCAGA | 108 | Elongation factor 1-delta 1 |
|  | R: TATCATCCTTCGAGGCTTGG |  |  |
| NbHsp17.3 | F: CACTCCACTGCTCCACACTC | 178 | 17.3 kDa class II heat shock protein |
|  | R: CAACCCTGGCATGTCTACAA |  |  |
| NbIRT1 | F: CGATTGCCTCAGGTCAGACT | 170 | Fe(2+) transport protein 1 (similar to) |
|  | R: ACTCCATCTTCCCGTTTCCT |  |  |
| NbMBF1C | F: GCCAAAGAAAGCACAAGACC | 132 | Multiprotein-bridging factor 1c |
|  | R: ACGACAGCGGAAGTAGCAGT |  |  |
| NbPAP1 | F: TCAGATGGAGTCTTGGCTCA | 167 | Plastid lipid-associated protein 1 |
|  | R: TAACAGACGCCACTTTCCAG |  |  |
| NbPNO1 | F: ACGCTTTCATGCTAGGGTTC | 155 | RNA-binding protein pno1 |
|  | R: CTTTCCTCCTTTCCCTGACA |  |  |
| NbPPCK | F: GGTGAAACTCCAACGGAGAC | 167 | Phosphoenolpyruvate carboxylase kinase |
|  | R: CATGGGTGTCTCAGGACTTG |  |  |
| NbPR1 | F: GGATGCCCATAACACAGCTC | 150 | Pathogenesis-related protein 1A |
|  | R: GCTAGGTTTTCGCCGTATTG |  |  |
| NbPR2 | F: ACAACAGTGCCGATGTCTCA | 174 | Glucan endo-1,3-beta-glucosidase |
