# Supplementary material for: The Defense Response of Nicotiana benthamiana to Peanut Stunt Virus Infection in the Presence of Symptom Exacerbating Satellite RNA
Source: Viruses. 2018 Aug 23;10(9):449. doi: 10.3390/v10090449 (PMC6165542; doi:10.3390/v10090449)
Supplement: Supplementary file 1 [file viruses-10-00449-s001.zip › Supplementary Files/Suppl. table S1.docx]

**Table S1.** Primers used in this study to satRNA synthesis, viral RNAs detection and virus accumulation assessment

| Primer name | Primer sequence (5’-3’) | Amplicon length [bp] | Function |
| --- | --- | --- | --- |
| T7satmod | ATGTAATACGACTCACTATAG  GTTTTGTTTTGTCGGGAGTC | 414 | satRNA synthesis (T7 promoter sequence underlined) (Obrępalska-Stęplowska, 2010) |
| satP3 | TCGACGGGGGTCGTGTAGGAGCTAAAG |  |  |
| gT7G1 | ATGTAATACGACTCACTATAGGTTTTATCACGAGCGTAC | 3384 | PSV-G RNA1 synthesis (T7 promoter sequence underlined) (Obrępalska-Stęplowska, 2010) |
| n3G1Pi2 | TCACGCGTGGTCTCCTTATGGAACCCT |  |  |
| gT7G2 | ATGTAATACGACTCACTATAGGTTTTATCAAGAGCCTACG | 3011 | PSV-G RNA2 synthesis (T7 promoter sequence underlined) (Obrępalska-Stęplowska, 2010) |
| n3TtAg3iG2 | TCACTAGTGGTCTCCTATGGAACCCA |  |  |
| gT7G3 | ATGTAATACGACTCACTATAGGTTTTACCAACCAGGAATC | 2215 | PSV-G RNA3 synthesis (T7 promoter sequence underlined) (Obrępalska-Stęplowska, 2010) |
| n3tG3 | TCACTAGTGGTCTCCTTATGGAACCCT |  |  |
| PSVCP | 1: TACCTTTTGGGTTCAATTCC | 918 | PSV detection (Obrepalska-Steplowska *et al.*, 2008) |
|  | 2: GACTGACCATTTTAGCCG |  |  |
| Sat | 1: GTTTTGTTTTGTCGGGAG | 393 | satRNA detection (Obrepalska-Steplowska *et al.*, 2008) |
|  | 2: GGGTCGTGTAGGAGC |  |  |
| PSVq1 | F: CTTCTGCCCTCGTTGATAAAG | 132 | Detection of PSV 1a protein ORF in real-time PCR (Obrępalska-Stęplowska, 2010) |
|  | R: CATACCGATTTCGAATCACTT |  |  |
| PSVq2a | F: CTTCTAGGTATCCCCGTAAG | 205 | Detection of PSV 2a protein ORF in real-time PCR (Obrępalska-Stęplowska, 2010) |
|  | R: CAAGCACATTGATACCCTATC |  |  |
| PSVq2b | F: CTCmTATCCTCCCAGCTAyAC | 78 | Detection of PSV 2b protein ORF in real-time PCR (Obrępalska-Stęplowska, 2010) |
|  | R: GAATAACTrCCCTCACACCAC |  |  |
| PSVq3a | F: CTAGTCGGACTTTAACACAAC | 247 | Detection of PSV 3a protein ORF in real-time PCR (Obrępalska-Stęplowska, 2010) |
|  | R: ACGCTCATATATCCCTTAGAC |  |  |
| PSVqCP | F: ACACATACACTTCGTTGGATG | 107 | Detection of PSV coat protein ORF in real-time PCR (Obrępalska-Stęplowska, 2010) |
|  | R: CCTCwTCTTCGGAAATTCAG |  |  |
| PARNA | 1: GGGAGGGCGGGCGTTCGTAGTG | 194 | satRNA detection in real-time PCR (Obrępalska-Stęplowska, 2010) |
|  | 2: GCCGTGGCCTTTCGTGGTC |  |  |
| NbEF1a | F: CACCATTGATATTGCCTTGTG | 91 | EF1α amplification in real-time PCR |
|  | R: GTTCTTGATAAAGTCCCTGTG |  |  |
| NbAct | A: GTGAAGGAGAAGTTGGCTTAC | 145 | β-actin amplification in real-time PCR (Obrępalska‐Stęplowska *et al.*, 2013) |
|  | 2: CTTCTGGGCAGCGGAATCTC |  |  |

**Obrepalska-Steplowska A, Budziszewska M, Pospieszny H**. 2008. Complete nucleotide sequence of a Polish strain of Peanut stunt virus (PSV-P) that is related to but not a typical member of subgroup I. *Acta Biochinica Polonica* **55**, 731-739.

**Obrępalska-Stęplowska A**. 2010. Rola satRNA w patogenezie szczepów wirusa karłowatości orzecha ziemnego (PSV) występujących w Polsce. *Rozprawy Naukowe Instytutu Ochrony Roślin* **Zeszyt 22**, 1-110.

**Obrępalska‐Stęplowska A, Wieczorek P, Budziszewska M, Jeszke A, Renaut J**. 2013. How can plant virus satellite RNAs alter the effects of plant virus infection? A study of the changes in the Nicotiana benthamiana proteome after infection by Peanut stunt virus in the presence or absence of its satellite RNA. *Proteomics* **13**, 2162-2175.
