# Supplementary material for: The Defense Response of Nicotiana benthamiana to Peanut Stunt Virus Infection in the Presence of Symptom Exacerbating Satellite RNA
Source: Viruses. 2018 Aug 23;10(9):449. doi: 10.3390/v10090449 (PMC6165542; doi:10.3390/v10090449)
Supplement: Supplementary file 1 [file viruses-10-00449-s001.zip › Supplementary Files/Supplementary Table S5.docx]

**Table S5.** Top 15 most affected pathways involving DEGs in PSV-G and PSV-G+satRNA infected plants

| **Down-regulation** | | **Up-regulation** | |
| --- | --- | --- | --- |
| **PSV-G** | **PSV-G+satRNA** | **PSV-G** | **PSV-G+satRNA** |
| \| ko01100 \| Metabolic pathways (50) \| \| --- \| --- \| \| ko01110 \| Biosynthesis of secondary metabolites (18) \| \| ko00195 \| Photosynthesis (13) \| \| ko01130 \| Biosynthesis of antibiotics (7) \| \| ko01200 \| Carbon metabolism (6) \| \| ko01120 \| Microbial metabolism in diverse environments (6) \| \| ko00190 \| Oxidative phosphorylation (5) \| \| ko03010 \| Ribosome (5) \| \| ko04070 \| Phosphatidylinositol signaling system (5) \| \| ko00196 \| Photosynthesis - antenna proteins (4) \| \| ko00564 \| Glycerophospholipid metabolism (4) \| \| ko04144 \| Endocytosis (4) \| \| ko04141 \| Protein processing in endoplasmic reticulum (3) \| \| ko04152 \| AMPK signaling pathway (3) \| \| ko00710 \| Carbon fixation in photosynthetic organisms (3) \| | \| ko01100 \| Metabolic pathways (190) \| \| --- \| --- \| \| ko01110 \| Biosynthesis of secondary metabolites (101) \| \| ko01130 \| Biosynthesis of antibiotics (45) \| \| ko00195 \| Photosynthesis (35) \| \| ko03010 \| Ribosome (33) \| \| ko01120 \| Microbial metabolism in diverse environments (32) \| \| ko01200 \| Carbon metabolism (30) \| \| ko01230 \| Biosynthesis of amino acids (17) \| \| ko00190 \| Oxidative phosphorylation (16) \| \| ko00860 \| Porphyrin and chlorophyll metabolism (16) \| \| ko00500 \| Starch and sucrose metabolism (16) \| \| ko00630 \| Glyoxylate and dicarboxylate metabolism (13) \| \| ko00010 \| Glycolysis / Gluconeogenesis (11) \| \| ko00520 \| Amino sugar and nucleotide sugar metabolism (11) \| \| ko00710 \| Carbon fixation in photosynthetic organisms (11) \| | \| ko03010 \| Ribosome (39) \| \| --- \| --- \| \| ko01100 \| Metabolic pathways (36) \| \| ko01110 \| Biosynthesis of secondary metabolites (20) \| \| ko03030 \| DNA replication (9) \| \| ko03040 \| Spliceosome (8) \| \| ko00230 \| Purine metabolism (7) \| \| ko01130 \| Biosynthesis of antibiotics (7) \| \| ko03013 \| RNA transport (7) \| \| ko03430 \| Mismatch repair (7) \| \| ko03420 \| Nucleotide excision repair (7) \| \| ko03440 \| Homologous recombination (6) \| \| ko04141 \| Protein processing in endoplasmic reticulum (6) \| \| ko04075 \| Plant hormone signal transduction (6) \| \| ko00240 \| Pyrimidine metabolism (6) \| \| ko01230 \| Biosynthesis of amino acids (5) \| | \| ko01100 \| Metabolic pathways (165) \| \| --- \| --- \| \| ko01110 \| Biosynthesis of secondary metabolites (93) \| \| ko03010 \| Ribosome (63) \| \| ko01130 \| Biosynthesis of antibiotics (47) \| \| ko01120 \| Microbial metabolism in diverse environments (41) \| \| ko01200 \| Carbon metabolism (28) \| \| ko03040 \| Spliceosome (27) \| \| ko04141 \| Protein processing in endoplasmic reticulum (26) \| \| ko03013 \| RNA transport (26) \| \| ko01230 \| Biosynthesis of amino acids (26) \| \| ko04626 \| Plant-pathogen interaction (20) \| \| ko00190 \| Oxidative phosphorylation (17) \| \| ko00270 \| Cysteine and methionine metabolism (17) \| \| ko03018 \| RNA degradation (15) \| \| ko03008 \| Ribosome biogenesis in eukaryotes (15) \| |
