# Supplementary figures and images for: The Defense Response of Nicotiana benthamiana to Peanut Stunt Virus Infection in the Presence of Symptom Exacerbating Satellite RNA
Source: Viruses. 2018 Aug 23;10(9):449. doi: 10.3390/v10090449 (PMC6165542; doi:10.3390/v10090449)

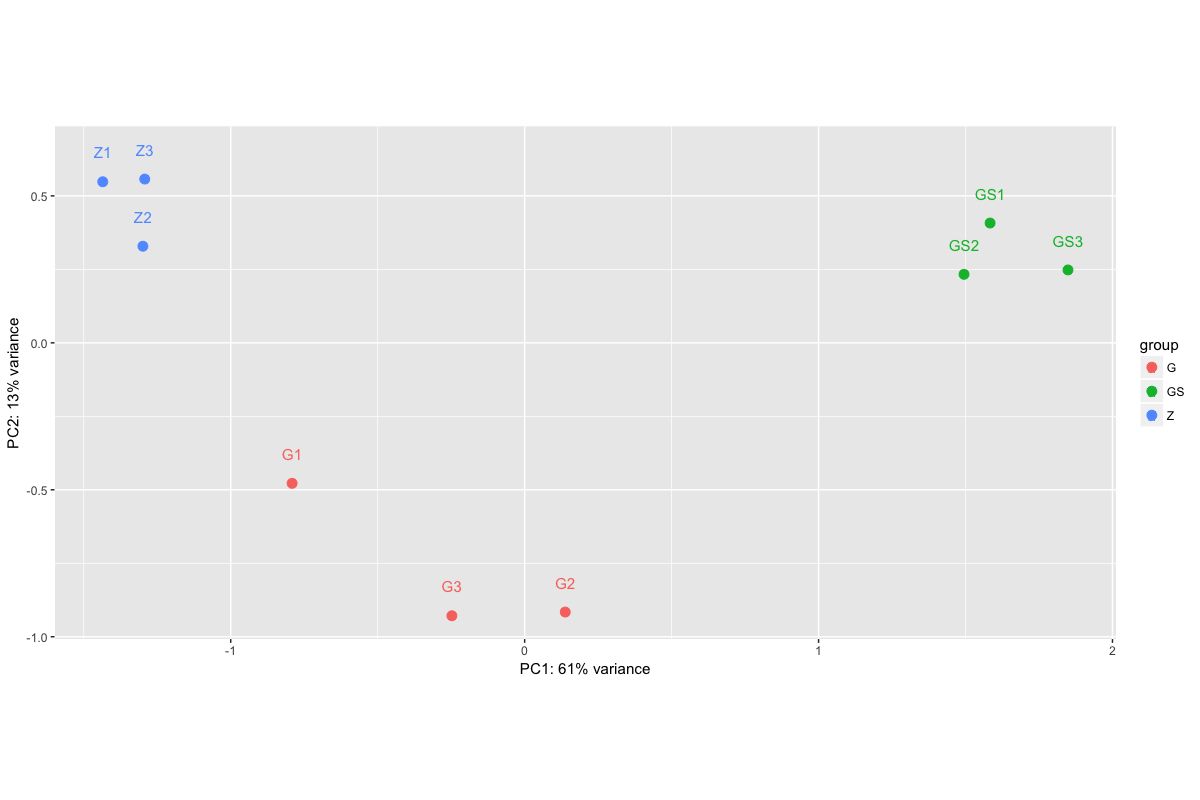

Supplement: Supplementary file 1 [file viruses-10-00449-s001.zip › Supplementary Files/Supplemental Figure 1.jpg]
