# Supplementary material for: The Defense Response of Nicotiana benthamiana to Peanut Stunt Virus Infection in the Presence of Symptom Exacerbating Satellite RNA
Source: Viruses. 2018 Aug 23;10(9):449. doi: 10.3390/v10090449 (PMC6165542; doi:10.3390/v10090449)
Supplement: Supplementary file 1 [file viruses-10-00449-s001.zip › Supplementary Files/Supplementary Table S4.doc]

**Table S4**. Prevailing terms among commonly up-regulated or down-regulated DEGs in plants infected by PSV-G and PSV-G+satRNA

| Direction of changes in gene expression | Biological process | Cellular component | Molecular function |
| --- | --- | --- | --- |
| Most abundant up-regulated DEGs in PSV-G and PSV-G+satRNA infected plants | translation, ribosome biogenesis, RNA metabolism process, response to stress and other stimuli, transport | nucleus, cytosol, and constituting integral component of membranes | RNA binding, protein binding, ion binding, transferase and hydrolase activity |
| Most abundant down-regulated DEGs in PSV-G and PSV-G+satRNA infected plants | oxidation-reduction process, photosynthesis, transport, nitrogen compound biosynthetic process, cellular lipid metabolism process | chloroplast structures | oxidoreductase, transferase, hydrolase activity, protein and nucleic acid binding |
